# Lymphocyte subsets in the peripheral blood are disturbed in systemic sclerosis patients and can be changed by immunosuppressive medication

**DOI:** 10.1007/s00296-021-05034-8

**Published:** 2021-10-25

**Authors:** Michael Gernert, Hans-Peter Tony, Eva Christina Schwaneck, Ottar Gadeholt, Matthias Fröhlich, Jan Portegys, Patrick-Pascal Strunz, Marc Schmalzing

**Affiliations:** 1 Department of Medicine II, Rheumatology and Clinical Immunology, University Hospital of Würzburg, Oberdürrbacher Str. 6, 97080 Würzburg, Germany; 2 Asklepios Klinik Altona, Rheumatology and Clinical Immunology, Paul-Ehrlich-Straße 1, 22763 Hamburg, Germany; 3 Rheumatologische Schwerpunktpraxis Würzburg, Haugerpfarrgasse 7, 97070 Würzburg, Germany

**Keywords:** Systemic sclerosis, Scleroderma, Memory B cells, B cell culture, Cytokines, γδ T cells, Immunophenotyping, Mycophenolate

## Abstract

Systemic sclerosis (SSc) is a severe chronic disease with a broad spectrum of clinical manifestations. SSc displays disturbed lymphocyte homeostasis. Immunosuppressive medications targeting T or B cells can improve disease manifestations. SSc clinical manifestations and immunosuppressive medication in itself can cause changes in lymphocyte subsets. The aim of this study was to investigate peripheral lymphocyte homeostasis in SSc with regards to the immunosuppression and to major organ involvement. 44 SSc patients and 19 healthy donors (HD) were included. Immunophenotyping of peripheral whole blood by fluorescence-activated cell sorting was performed. Cytokine secretions of stimulated B cell cultures were measured. SSc patients without immunosuppression compared to HD displayed lower γδ T cells, lower T helper cells (CD3^+^/CD4^+^), lower transitional B cells (CD19^+^/CD38^++^/CD10^+^/IgD^+^), lower pre-switched memory B cells (CD19^+^/CD27^+^/IgD^+^), and lower post-switched memory B cells (CD19^+^/CD27^+^/IgD^−^). There was no difference in the cytokine production of whole B cell cultures between SSc and HD. Within the SSc cohort, mycophenolate intake was associated with lower T helper cells and lower NK cells (CD56^+^/CD3^−^). The described differences in peripheral lymphocyte subsets between SSc and HD generate further insight in SSc pathogenesis. Lymphocyte changes under effective immunosuppression indicate how lymphocyte homeostasis in SSc might be restored.

**Supplementary Information:**

The online version contains supplementary material available at 10.1007/s00296-021-05034-8.

## Background

Systemic sclerosis (SSc) is characterized by pathologic fibrosis of the skin and internal organs, and by vasculopathy with Raynaud’s phenomenon and digital ulcers. In early disease states, inflammatory manifestations such as arthritis may occur [1]. The pathogenesis of SSc is not fully understood. Lymphocyte characterizations have revealed altered T cell subsets [2] and B cell subsets [3, 4] with secretion of pro-fibrotic cytokines [5, 6]. Within the T cell compartment, lymphocytes expressing the γδ T cell receptor (γδ T cells) are widely investigated in autoimmune diseases and are disturbed in SSc [7]. γδ T cells participate in innate and acquired immune functions: As part of the first line of defense, they expand in the initial phase of infections. They produce interleukin (IL)-17 and thereby attract neutrophils [8–10]. Also, γδ T cells support B cell antibody class switching and dendritic cell maturation [11–13]. In the later immune response, γδ T cells downregulate activated macrophages and other T cells and promote tissue repair [11, 14, 15].

Within the B cell compartment, memory B cells are of special interest in autoimmune diseases. Memory B cells in the peripheral blood are mostly characterized by cluster of differentiation (CD) 27-expression. They can be categorized in pre- and post-switched memory B cells according to their immunoglobulin (Ig) D-expression (CD19^+^/CD27^+^/IgD^+^ and CD19^+^/CD27^+^/IgD^−^, respectively) and show hypermutation in the immunoglobulin variable-region genes [16, 17]. Double negative (DN) B cells (CD19^+^/CD27^−^/IgD^−^), although lacking CD27-expression, show somatic hypermutation of the B cell receptor and are, therefore, attributed to the memory compartment [18]. DN B cells play a pathogenic role in autoimmune diseases, as they are enhanced and activated in systemic lupus erythematosus (SLE) and rheumatoid arthritis (RA) [18–20].

Both, the T cell and the B cell compartment are targeted effectively by immunosuppressive medications in SSc. However, data on immunosuppressive treatment in SSc is limited. The European League against Rheumatism (EULAR) recommendations for the treatment of SSc [21] include methotrexate in early disease stages [22]. Cyclophosphamide [23], rituximab [24, 25], and mycophenolate [26] showed efficacy in SSc interstitial lung disease. Autologous hematopoietic stem cell transplantation (aHSCT) has shown superiority compared to cyclophosphamide in three randomized controlled trials (ASSIST [27], ASTIS [28] and SCOT [29]).

It is known that immunosuppressive medication changes lymphocyte subsets in SSc [30, 31]. Conversely, alterations in lymphocyte subsets influence effectiveness and adverse event rates of immunosuppressive medication [32]. Furthermore, disease stage influences the lymphocyte composition in SSc [33]. Therefore, the mode of immunosuppressive medication and major organ involvement has to be taken into account, when lymphocytes of SSc patients are analyzed.

The aim of the present study was to characterize differences in lymphocyte subsets and B cell function of SSc patients compared to healthy controls taking the immunosuppressive medication and major organ involvement into consideration.

## Patients and methods

### Patients and healthy donors

44 patients fulfilling the American College of Rheumatology (ACR)/EULAR criteria [34] for SSc and 19 healthy donors (HD) were included between March 2015 and September 2018 in this study. Clinical data was collected in electronic files (EMIL by itc‐ms.de, Marburg, Germany and SAP SE, Walldorf, Germany). SSc patients (whole cohort = SSc_total_) were grouped (a) according to their immunosuppressive (immunomodulatory) medication at the time of blood collection (SSc patients without immunosuppressive medication (SSc_noIS_), intake of methotrexate, azathioprine, hydroxychloroquine, or mycophenolate (SSc_+MMF_)), (b) according to the extent of skin involvement (limited cutaneous form (lcSSc) or diffuse cutaneous form (dcSSc)), (c) according to SSc disease duration (under (or equal to) 5 years or over 5 years), and (d) according to presence of lung fibrosis (lung fibrosis on computed tomography present or not).

### Immunophenotyping for characterization of lymphocyte percentages

**Fig. 1:**
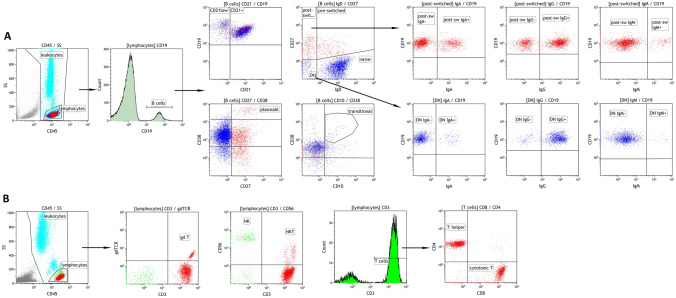
Lymphocyte characterization by flow cytometry. Gating strategy with representative blots **A** for B cell subsets and **B** for T cell subsets and NK cells. *Post-sw* post-switched memory B cells, *plasmabl* plasmablasts, *DN* double negative B cells, *gd T* γδ T cells

Peripheral blood was collected in EDTA tubes and immediately processed. 10 µl of each antibody were applied and incubated for 15 min at room temperature. The following antibodies were used in different combinations for fluorescence activated cell sorting (FACS): CD3-PC7, CD4-FITC, CD8-ECD, CD14-PE, CD19-PC7 and -ECD, CD20-APC750, CD27-ECD, CD38-PC5.5, CD45-Krome-Orange and -FITC, CD56/16-PC5, γδTCR-PE (each Beckman Coulter, Krefeld, Germany), CD21-PB (Exbio, Prague, Czech Republic), IgM-APC (BioLegend, San Diego, CA), CD10-PE and IgD-FITC (both BD Biosciences, San Jose, CA). VersaLyse together with IOTest3 Fixative Solution (2 ml, ratio 2:1; both Beckman Coulter, Krefeld, Germany) were used for erythrocyte lysing. Cells were centrifuged at 300 relative centrifugal force (RCF) for 15 min and the pellet was resuspended in 2 ml phosphate buffered saline (+ 1% fecal calf serum). Another centrifugation step at 300 RCF for 15 min followed with resuspensation of the pellet in 300 µl phosphate buffered saline (+ 1% fecal calf serum). To detect lymphocytes, forward scatter vs side scatter and CD45 staining was plotted and at least 3000 events were collected within the lymphocyte gate. Within the lymphocyte gate T cells were defined as CD3^+^ events, T helper cells as CD3^+^/CD4^+^, cytotoxic T cells as CD3^+^/CD8^+^, NK cells as CD3^−^/CD56/16^+^, NKT cells as CD3^+^/CD56/16^+^, γδ T cells as gamma/delta T cell receptor positive lymphocytes. The T cell panel was measured with a Cytomics FC500MCL cytometer (Beckman Coulter, Krefeld, Germany). B cells were defined as CD19^+^ events within the lymphocyte gate. Within the B cell compartment CD38^++^/CD10^+^/IgD^+^ events were defined as transitional B cells, CD27^−^/IgD^+^ events as naïve B cells, CD27^+^/IgD^+^ events as pre-switched memory B cells, CD27^+^/IgD^−^ events as post-switched memory B cells and CD27^−^/IgD^−^ events as double negative (DN) B cells. CD19^+^/CD21^−^ events were defined as CD21^low^ B cells. Further gating was applied on post-switched memory B cells and on DN B cells to describe the distribution of surface IgA, IgG and IgM. The B cell panels were measured with a Navios 3L10c cytometer (Beckman Coulter, Krefeld, Germany). FACS blots were generated with Kaluza software v1.2 (Beckman Coulter, Krefeld, Germany). The gating strategy is shown in Fig. 1.

### Differential blood count to calculate absolute lymphocyte numbers

25 µl of EDTA-anticoagulated whole blood were measured in a XN-550 automated hematology analyzer (Sysmex, Kobe, Japan) to determine the absolute lymphocyte numbers (i.e., lymphocytes per microliter whole blood). The lymphocyte number was multiplied with the respective cell percentages obtained in the FACS analyses to calculate absolute numbers of lymphocyte subsets.

### Peripheral blood mononuclear cells (PBMCs) preparation and B cell enrichment

Ficoll-Paque Plus separation (GE Healthcare, Munich, Germany) was used according to the manufacturer’s instructions to isolate PBMCs out of 15–20 ml peripheral blood from EDTA-tubes. PBMCs were stored in liquid nitrogen before further processing. Magnetic cell sorting (MACS) with CD19 monoclonal antibody-coupled microbeads (Miltenyi Biotec, Bergisch Gladbach, Germany) was performed. PBMCs were twice positively selected (i.e., two consecutive columns) for CD19 to achieve a purity of more than 87% of B cells for the B cell cultures.

### B cells cultures and cytokine measurement

B cell cultures with 1–2 × 10^5^ cells per sample were incubated over 24 h with 10 µg/ml cytosine guanine dinucleotide (CpG ODN 2006, InvivoGen, Toulouse, France) at 37 °C. Supernatants were collected and cytokines were measured with cytometric bead arrays (CBA flex set; BD bioscience, San Jose, CA) using a LSR II cytometer (BD bioscience, San Jose, CA). FCAP array software (BD bioscience, San Jose, CA) was used to calculate cytokine concentrations.

### Statistical analysis

Shapiro–Wilk tests were used to test for normal distribution. As normal distribution was mostly absent, medians with interquartile ranges (IQR) were shown. For continuous variables Wilcoxon signed-rank tests were performed to detect differences between paired groups and Mann–Whitney *U* tests for unpaired groups. For categorical variables Fisher’s exact tests were used. Calculations were done with SPSS Statistics v 26.0 (IBM, Armonk, New York). For data collection Excel (Microsoft, Redmond, Washington) was used. Figures were grouped using Photoshop (Adobe, San Jose, California). Two-tailed *P *values less than 0.05 were considered significant.

## Results

### Patients’ characteristics

44 SSc patients participated in this study. 36 patients (81.8%) were female, mean age was 58.1 years (range 22–82 years). Mean disease duration before blood collection was 9.4 years (range 3 months–40 years). 11 patients (25.0%) had a diffuse cutaneous form. 42 patients (95.5%) were anti-nuclear antibody (ANA) positive, 17 patients (38.6%) were Scl-70 antibody positive, 18 patients (40.9%) were centromere antibody positive. 27 patients (61.4%) had pulmonary fibrosis, and 3 patients (6.8%) had cardiac involvement of SSc proven by myocardial biopsy or MRI. 6 Patients (13.6%) had pulmonary arterial hypertension. Mean modified Rodnan skin score (mRSS) was 6.3 (range 0–37). Patients who had undergone an autologous hematopoietic stem cell transplantation in the past were not included in this study. At the time of sample collection, the 44 SSc patients (SSc_total_ cohort) received the following immunosuppressive medications: No immunosuppressive medication 17 patients (38.6%), methotrexate 7 patients (15.9%), azathioprine 1 patient (2.3%), hydroxychloroquine 7 patients (15.9%), and mycophenolate 12 patients (27.3%) (Table 1). Each immunosuppressive medication was taken at least for 3 months before blood collection. 19 healthy persons (HD) donated blood to serve as comparison group, 13 of them (68.4%) were female and their mean age was 49.7 years (range 23–79 years).

**Table 1:** Characteristics of the SSc patients

Characteristics	Values
Female, *n* (%)	36/44 (81.8)
Age, mean (range), years	58.1 (22–82)
Disease duration, mean (range), years	9.4 (3 months–40 years)
Diffuse cutaneous form, *n* (%)	11/44 (25.0)
Anti-nuclear antibody positivity, *n* %	42/44 (95.5)
Anti-Scl-70 antibody positivity, *n* (%)	17/44 (38.6)
Anti-Centromere antibody positivity, *n* (%)	18/44 (40.9)
mRSS, mean (range), points	6.3 (0–37)
Pulmonary fibrosis on thoracic computed tomography, *n* (%)	27/44 (61.4)
Cardiac involvement, *n* (%)	3/44 (6.8)
Pulmonary arterial hypertension, *n* (%)	6/44 (13.6)
Immunosuppressive medication	
None, *n* (%)	17/44 (38.6)
Mycophenolate, *n* (%)	12/44 (27.3)
Methotrexate, *n* (%)	7/44 (15.9)
Hydroxychloroquine, *n* (%)	7/44 (15.9)
Azathioprine, *n* (%)	1/44 (2.3)

*mRSS* modified Rodnan skin score, *SSc* systemic sclerosis

### Altered memory B cells, transitional B cells, T helper cells, and γδ T cells in SSc compared to HD

To exclude the influence of immunosuppressive medication on lymphocyte subsets, SSc patients without immunosuppressive medication (SSc_noIS_, *n* = 17) were compared to HD. Within the B cell compartment SSc patients had lower pre-switched memory B cell numbers compared to HD (10.0 [4.8–20.8]/µl vs 36.1 [12.5–53.2]/µl, *P* = 0.007), lower post-switched memory B cells (10.9 [6.7–20.2]/µl vs 29.1 [17.3–44.4]/µl, *P* = 0.016), and lower transitional B cells (2.2 [1.3–9.1]/µl vs 6.4 [4.0–13.2, *P* = 0.030). Surface expressions of IgA, IgG and IgM on post-switched memory B cells and double negative B cells were analyzed. Both, IgA^+^ and IgG^+^ post-switched memory B cells were lower in SSc patients compared to HD (CD19^+^/CD27^+^/IgD^−^/IgA^+^: 3.0 [1.7–8.5]/µl vs 10.3 [4.9–14.7, *P* = 0.022. CD19^+^/CD27^+^/IgD^−^/IgG^+^: 3.7 [1.9–5.8]/µl vs 11.0 [3.1–25.8, *P* = 0.018).

**Fig. 2:**
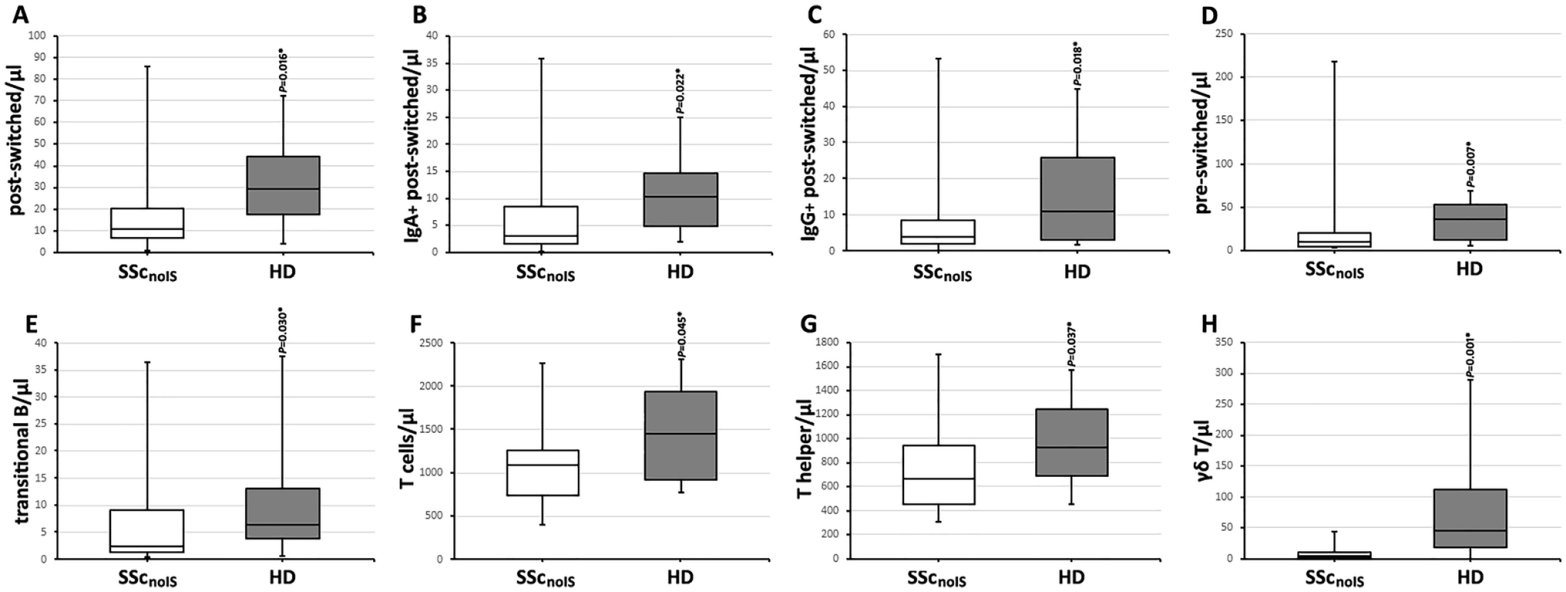
Comparison of lymphocyte subsets of SSc patients without immunosuppressive medication (SSc_noIS_, white boxes,* n* = 17) with healthy donors (HD, grey boxes, *n* = 19). SSc patients had significant lower post-switched memory B cells (**A**) and within the post-switched memory B cells lower IgA^+^ (**B**) and IgG^+^ cell numbers (**C**) compared to HD. Pre-switched memory B cells (**D**) and transitional B cells (**E**) were also lower in SSc_noIS_. Lower T cells (**F**), lower T helper cell numbers (**G**), and γδ T cell numbers (**H**) were present in SSc_noIS_ compared to HD. Boxplots show medians with 25th and 75th percentiles, whiskers indicate minimums and maximums, respectively. *Significant differences in a Mann–Whitney *U* test, *P* < 0.05

Within the T cell compartment SSc patients displayed lower T cells (1085.7 [739.6–1254.7]/µl vs 1458.1 [920.4–1943.7]/µl, *P* = 0.045), lower T helper cell numbers (670.6 [453.0–947.1]/µl vs 922.5 [693.1–1244.9]/µl, *P* = 0.037) and lower γδ T cell numbers (4.9 [1.0–10.5]/µl vs 44.8 [18.5–111.5]/µl, *P* = 0.001) compared to HD. In other lymphocyte subsets (NK cells, NKT cells, cytotoxic T cells) no significant differences between SSc_noIS_ and HD were present. Table 2 shows the lymphocyte numbers (and the lymphocyte percentages obtained from FACS to calculate lymphocyte numbers). Figure 2 shows significant differences.

**Table 2:** Lymphocyte subsets of SSc patients without immunosuppressive medication (SSc_noIS_) compared to HD in percentages within the lymphocyte (subset) gates and in absolute numbers (/µl)

	Percentages % (IQR)		Absolute numbers/µl (IQR)	
	SSc_noIS_	HD	SSc_noIS_	HD
Lymphocytes	21.3 (14.8–26.8)	34.5 (18.8–42.7)*	1670.0 (1205.0–1855.0)	1870.0 (1510.0–2510.0)
% within the lymphocyte gate				
T cells CD3^+^	62.0 (56.0–72.2)	71.4 (65.3–78.2)*	1085.7 (739.6–1254.7)	1458.1 (920.4–1943.7)*
NK cells CD56/CD16^+^	18.2 (13.5–25.5)	11.6 (8.8–18.4)*	285.5 (231.0–408.5)	242.0 (170.5–318.6)
NKT cells CD3^+^/CD56/CD16^+^	2.3 (1.1–6.8)	4.0 (2.1–7.7)	32.2 (10.4–124.0)	70.1 (28.6–190.6)
Cytotoxic T cells CD3^+^/CD8^+^	15.8 (11.5–19.5)	18.9 (14.9–23.3)	271.6 (151.5–362.1)	376.8 (226.4–519.2)
T helper cells CD3^+^/CD4^+^	42.8 (38.8–53.3)	49.5 (43.4–53.8)	670.6 (453.0–947.1)	922.5 (693.1–1244.9)*
CD4^+^/CD8^+^ ratio			2.3 (2.0–4.6)	2.6 (1.9–3.4)
γδ T cells	0.4 (0.2–1.1)	2.0 (1.4–5.1)*	4.9 (1.0–10.5)	44.8 (18.5–111.5)*
Total B cells CD19^+^	12.1 (8.4–20.2)	10.2 (8.6–15.2)	245.5 (112.9–309.8)	201.0 (165.7–308.6)
% within the CD19^+^ compartment				
Transitional B cells CD38^++^/CD10^+^/IgD^+^	1.5 (0.8–3.2)	3.0 (1.8–5.1)*	2.3 (1.3–9.1)	6.4 (3.9–13.2)*
Plasmablasts CD38^+^/CD27^++^/IgD^−^	0.7 (0.5–1.3)	0.8 (0.5–1.4)	1.3 (1.0–2.3)	1.9 (1.3–3.1)
CD21^low^ B cells CD19^+^/CD21^−^	9.8 (4.8–15.8)	6.3 (3.5–7.5)*	16.5 (6.3–46.7)	12.4 (8.4–19.1)
Naïve B cells CD27^−^/IgD^+^	82.8 (72.9–90.3)	69.8 (59.2–81.1)*	151.1 (93.9–244.0)	149.5 (111.1–215.4)
Pre-switched memory B cells CD27^+^/IgD^+^	5.1 (3.1–11.0)	13.0 (7.0–19.5)*	10.0 (4.8–20.8)	36.1 (12.5–53.2)*
Post-switched memory B cells CD27^+^/IgD^−^	8.0 (3.4–14.1)	12.9 (8.6–19.1)*	10.9 (6.7–20.2)	29.1 (17.3.44.4)*
Double negative B cells CD27^−^/IgD^−^	3.7 (2.4–4.7)	3.2 (2.5–4.2)	5.6 (4.0–10.0)	6.5 (4.9–10.3)
% within the post-switched memory B cell compartment				
CD27^+^/IgD^−^/IgA^+^	31.6 (24.4–40.1)	36.1 (28.4–44.7)	3.0 (1.7–8.5)	10.3 (4.9–14.7)*
CD27^+^/IgD^−^/IgG^+^	33.5 (14.3–50.6)	49.2 (40.4–58.8)	3.6 (1.9–5.8)	11.0 (3.1–25.8)*
CD27^+^/IgD^−^/IgM^+^	11.3 (4.6–15.2)	5.1 (4.2–7.1)*	1.0 (0.6–2.0)	1.6 (1.0–2.9)
Post-switched IgG^+^/IgA^+^ ratio			0.8 (0.4–1.7)	1.4 (0.9–2.0)
% within the double negative B cell compartment				
CD27^−^/IgD^−^/IgA^+^	21.4 (12.3–28.2)	22.0 (14.9–25.2)	1.1 (0.6–2.1)	1.1 (0.8–2.2)
CD27^−^/IgD^−^/IgG^+^	50.6 (24.5–68.2)	68.4 (59.0–72.7)	2.5 (0.9–5.1)	4.0 (2.2–8.1)
CD27^−^/IgD^−^/IgM^+^	14.6 (2.9–24.9)	2.6 (1.6–11.4)*	0.4 (0.2–2.4)	0.3 (0.1–0.4)
Double negative IgG^+^/IgA^+^ ratio			2.0 (1.1–4.6)	3.1 (2.4–5.3)

Shown are medians (IQR, interquartile range); *HD* healthy donors; *SSc* systemic sclerosis; The IgG^+^/IgA^+^ ratio is the calculated median of the patients’ individual ratios; *n*_SSc_ = 17; *n*_HD_ = 19; *significant (*P* < 0.05) in a Mann–Whitney *U* test comparing HD with SSc_noIS_

### DN B cells and post-switched memory B cells have a different IgG/IgA ratio

The ratio of surface IgG- and IgA-expression on DN B cells was high, but not different between SSc_noIS_ and HD (2.0 [1.1–4.6] vs 3.1 [2.4–5.3, *P* = 0.138). The IgG/IgA ratio on post-switched memory B cells was more balanced and not different between SSc_noIS_ and HD (0.8 [0.4–1.7] vs 1.4 [0.9–2.0, *P* = 0.208) (Table 2).

### Disease characteristics do not alter lymphocyte subsets in SSc

To analyze the effect of skin involvement on lymphocytes, lcSSc vs dcSSc was compared. Differences in the numbers of the lymphocyte subsets were not detected. Also no differences were seen when SSc patients with lung fibrosis were compared to patients without lung fibrosis. Patients with a long-lasting disease (over 5 years) did not have differences in their lymphocyte subsets comparted to patients with shorter disease duration.

### Mycophenolate intake correlates with lower T helper cells and NK cells in SSc

**Fig. 3:**
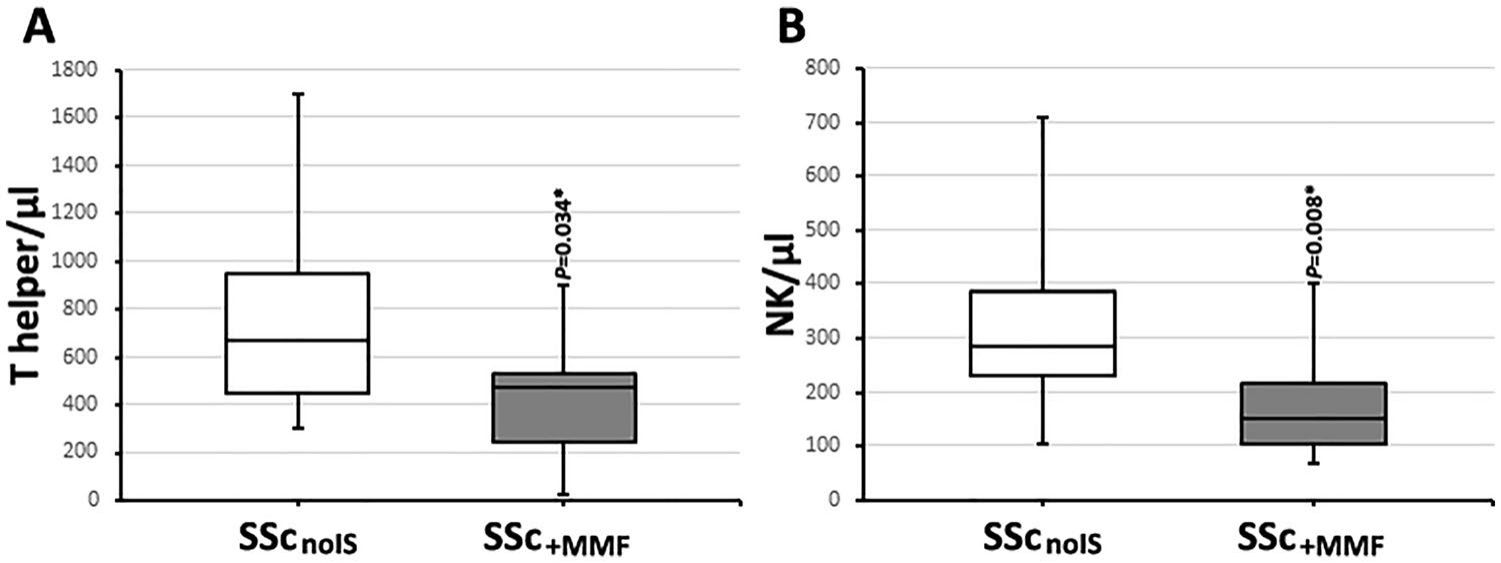
Comparison of lymphocytes subsets of SSc patients without immunosuppressive medication (SSc_noIS_, white boxes, *n* = 17) with SSc patients taking mycophenolate (SSc_+MMF_, grey boxes, *n* = 12). SSc_+MMF_ had significant lower T helper cells (**A**) and lower NK cells (**B**). Boxplots show medians with 25th and 75th percentiles, whiskers indicate minimums and maximums, respectively. *Significant differences in a Mann–Whitney *U* test, *P* < 0.05

To analyze the effect of mycophenolate on lymphocyte subsets SSc patients without any immunosuppressive medication (SSc_noIS_ = 17) were compared to SSc patients receiving mycophenolate (SSc_+MMF_, *n* = 12). SSc_+MMF_ patients had lower T helper cells (670.6 [453.0–947.1]/µl vs 470.3 [244.3–533.6]/µl, *P* = 0.034) and lower NK cells/µl (285.5 [231.0–408.5]/µl vs 150.3 [106.0–216.2]/µl, *P* = 0.008) (Fig. 3). Disease characteristics might influence the prescription of mycophenolate. In our cohort no differences in the prevalence of mycophenolate intake were seen between lsSSc vs dcSSc (*P* = 0.139) and between disease duration under (or equal to) 5 years vs over 5 years (*P* = 0.102). Patients with lung fibrosis took more often mycophenolate (11/27) compared to patients without lung fibrosis (1/16, *P* = 0.015). No differences in the lymphocyte subsets were seen in SSc patients taking methotrexate or hydroxychloroquine compared to SSc_noIS_.

### Comparison of cytokine productions in B cell cultures between HD and SSc

Cytokine concentrations in CpG-stimulated B cell cultures were detectable for IL-6, IL-10, IL-1β, tumor necrosis factor (TNF)-α and IL12/23(p40). Cytokine secretions were inducible upon CpG stimulation (Supplementary Figure S1). No differences in the median cytokine productions were seen comparing B cell cultures of SSc_noIS_ patients vs HD (IL-6: SSc 296.5 [171.3–493.2] pg/ml vs HD 301.2 [210.2–500.0] pg/ml, *P* = 0.754; IL-10: 1.0 [0.0–2.4] vs 1.2 [0.0–3.6] pg/ml, *P* = 0.639; IL-1β: 0.2 [0.0–3.2] vs 0.0 [0.0–0.7] pg/ml, *P* = 0.219; TNF-α: 14.2 [7.0–27.6] vs 20.1 [12.9–43.9] pg/ml, *P* = 0.165; IL12/23(p40): 0.0 [0.0–0.0] vs 0.0 [0.0–0.5] pg/ml, *P* = 0.471) (Supplementary Figure S2). Also no differences in the cytokine secretion in B cell cultures could be detected when immunosuppressive medication, extent of skin involvement, disease duration, or lung fibrosis were regarded for analysis.

## Discussion

In this study, we characterized the lymphocyte subsets of 44 SSc patients, with respect to their immunosuppressive medication and to major organ involvement in comparison to healthy controls. The reduction of γδ T cells, reduction of transitional B cells, reduction of pre-switched memory B cells and reduction of IgA^+^ and IgG^+^ post-switched memory B cells can be attributed to SSc per se, whereas the reduction of T helper cells and NK cells might be explained by mycophenolate intake.

Within the T cell compartment reduced γδ T cells in PBMCs of SSc (compared to HD) [33, 35] and in the peripheral blood [36] have been reported. In contrast, γδ T cells are elevated in skin lesions of SSc [37]. The lowest γδ T cells in SSc patients were reported when Scl-70 antibodies were present, a diffuse cutaneous form was present and in patients with short disease duration (less than 3 years) [7]. It seems that lower γδ T cells are associated with more aggressive and early forms of SSc.

We investigated the effect of immunosuppressive medications on lymphocyte subsets in SSc patients and could show that patients taking mycophenolate therapy had significant lower T helper cell and NK cell numbers. Among our SSc patients, those taking mycophenolate were the largest group (*n* = 12 of 44) and they were balanced concerning their extent of skin involvement (7 were lcSSc and 5 were dcSSc). This might be one possible reason for the detection of significant changes. All other immunosuppressive medications such as methotrexate or hydroxychloroquine were taken by fewer patients. Our findings match the expected mode of action of mycophenolate as it inhibits lymphocyte proliferation [38]. Inhibition of T helper cells in SSc patients seems reasonable, as T helper cells are known to participate in the pathogenesis of SSc [39, 40].

B cells in SSc exhibit an activated status, and contribute to SSc pathogenesis by production of profibrotic cytokines and autoantibodies [3]. So far there is little data available about the role of memory B cells in SSc pathogenesis. Other findings support our results, showing reduced total memory B cells (CD19^+^/CD27^+^) in SSc though activated with overexpression of CD19 and CD95 [41]. We detected reduced memory B cells in SSc with a significant reduction of pre-switched memory B cells and post-switched memory B cells compared to HD. Examination of IgA^+^ and IgG^+^ subgroups on post-switched memory B cells revealed significant reductions in both populations. Thereby an IgG/IgA ratio of 1.4 on post-switched memory B cells in HD is not different in SSc. In a cohort of RA patients the IgG/IgA ratio of post-switched memory B cells was similar to our SSc patients and not changed by immunosuppressive medication [19]. The reduced numbers of IgA + and IgG + post-switched memory B cells indicate an impaired adaptive immune response in SSc, the class switch process of B cells, however, seems to be intact. Another explanation for reduced memory B cells in SSc patients could be an augmented apoptosis of memory B cells [41].

The disturbance of memory B cell homeostasis in SSc is different from diseases, thought to be mainly antibody driven, such as RA and SLE. Patients with these diseases exhibit elevated DN memory B cells [18, 19, whereas in SSc DN memory B cell counts are not different to HD. DN B cells of our SSc patients exhibited an IgG/IgA ratio of 2.0 and HD of 3.1. A similar high IgG/IgA ratio is reported in RA [19]. The low switch to IgA may be inherent in DN B cells and might reflect their putative abortive or inadequate differentiation niche in autoimmune diseases as well as in healthy individuals. In concordance with this finding, CD27^−^ memory B cells have a lower rate of somatic hypermutation compared to CD27^+^ memory B cells [42]. It has been postulated that impaired germinal center reactions or extra-follicular reactions might be the reason for a suboptimal DN memory B cell maturation [43]. IgG^+^ DN memory B cells are increased in elderly people indicating they might be late memory or exhausted memory B cells [44].

Our results of unaltered cytokine productions in whole B cell cultures fit to the findings in the immunophenotyping, where no differences in whole B cell percentages and B cell numbers between SSc and HD were detected. To investigate the functional relevance of the alterations that were found by immunophenotyping in the pre-switched and post-switched memory B cell compartment, these cells have to be investigated separately in cell cultures. This could be addressed in future studies.

The described disturbances in B cell subsets may reflect pathogenic factors for SSc and might be one reason why targeting B cells is an effective treatment in SSc: Application of rituximab showed improvements of skin and lung manifestations of SSc [25, [45]. Rituximab causes a long-lasting depletion of the memory B cell compartment [46]. B cell depletion also causes changes in the T cell compartment with reduction of memory T helper cells [47]. But none of our SSc patients received rituximab.

Limitations of our study are the single center design and the singular blood collection per patient with a lack of follow-up values.

## Conclusion

SSc patients display a disturbed lymphocyte homeostasis, both, within the B cell compartment (with reduced pre-switched memory B cells and reduced IgA and IgG expressing post-switched memory B cells) and within the T cell compartment (with reduced γδ T cells and T helper cells). These changes may reflect SSc pathogenesis and might offer future therapeutic options.

## Supplementary Information

Below is the link to the electronic supplementary material.

Supplementary file 1. Figure S1. Cytokine secretions in B cell cultures are induced by toll-like receptor 9 stimulator CpG ODN. 35 samples (24 SSc patients and 11 healthy donors) were aliquoted. Three aliquots of each sample was treated with CpG (+CpG, grey boxes) for B cell stimulation and the mean compared to the mean of three aliquots of the unstimulated B cell culture (noCpG, white boxes). Significant increased cytokine concentrations were measured for: IL-6 (median 419.7 pg/ml [interquartile range 210.2–565.4 pg/ml] vs 19.9 [11.9–36.1] pg/ml, P < 0.001 ), IL-10 (1.5 [0.1–3.6] vs 0.0 [0.0–0.0] pg/ml, P < 0.001), IL-1β (0.0 [0.0–0.9] vs 0.0 [0.0–0.1] pg/ml, P = 0.024), TNF-α (18.8 [10.3–37.1] vs 0.0 [0.0–0.1] pg/ml, P < 0.001), IL12/23(p40) (0.0 [0.0–0.8] vs 0.0 [0.0–0.0] pg/ml, P = 0.003). Boxplots show medians with 25th and 75th percentiles, whiskers indicate minimums and maximums, respectively. * significant difference in a Wilcoxon signed-rank test, P < 0.05. (TIF 1792 KB)

Supplementary file 2. Figure S2. Cytokine secretions in B cell cultures of systemic sclerosis patients without immunosuppressive medication (SScnoIS, white boxes, n = 17) compared to healthy controls (HD, grey boxes; n = 19). No significant differences were seen in IL-6, IL-10, IL-1β, TNF-α, and IL12/23(p40) production. Boxplots show medians with 25th and 75th percentiles, whiskers indicate minimums and maximums, respectively. (TIF 1088 KB)
