# Supplementary figures and images for: Lymphocyte subsets in the peripheral blood are disturbed in systemic sclerosis patients and can be changed by immunosuppressive medication
Source: Rheumatol Int. 2021 Oct 25;42(8):1373–81. doi: 10.1007/s00296-021-05034-8 (PMC9287253; doi:10.1007/s00296-021-05034-8)

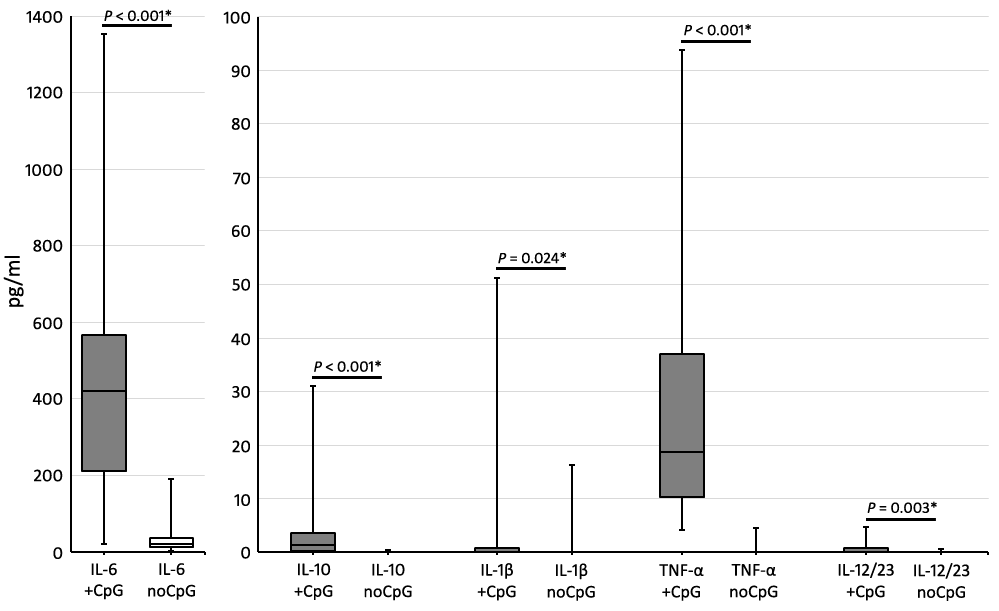

Supplement: Supplementary file 1 — Figure S1. Cytokine secretions in B cell cultures are induced by toll-like receptor 9 stimulator CpG ODN. 35 samples (24 SSc patients and 11 healthy donors) were aliquoted. Three aliquots of each sample was treated with CpG (+CpG, grey boxes) for B cell stimulation and the mean compared to the mean of three aliquots of the unstimulated B cell culture (noCpG, white boxes). Significant increased cytokine concentrations were measured for: IL-6 (median 419.7 pg/ml [interquartile range 210.2–565.4 pg/ml] vs 19.9 [11.9–36.1] pg/ml, P < 0.001 ), IL-10 (1.5 [0.1–3.6] vs 0.0 [0.0–0.0] pg/ml, P < 0.001), IL-1β (0.0 [0.0–0.9] vs 0.0 [0.0–0.1] pg/ml, P = 0.024), TNF-α (18.8 [10.3–37.1] vs 0.0 [0.0–0.1] pg/ml, P < 0.001), IL12/23(p40) (0.0 [0.0–0.8] vs 0.0 [0.0–0.0] pg/ml, P = 0.003). Boxplots show medians with 25th and 75th percentiles, whiskers indicate minimums and maximums, respectively. * significant difference in a Wilcoxon signed-rank test, P < 0.05. (TIF 1792 KB) [file 296_2021_5034_MOESM1_ESM.tif]

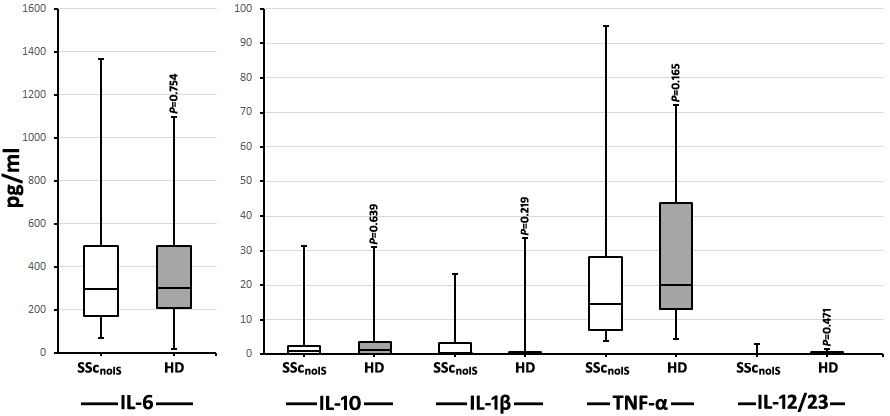

Supplement: Supplementary file 2 — Figure S2. Cytokine secretions in B cell cultures of systemic sclerosis patients without immunosuppressive medication (SScnoIS, white boxes, n = 17) compared to healthy controls (HD, grey boxes; n = 19). No significant differences were seen in IL-6, IL-10, IL-1β, TNF-α, and IL12/23(p40) production. Boxplots show medians with 25th and 75th percentiles, whiskers indicate minimums and maximums, respectively. (TIF 1088 KB) [file 296_2021_5034_MOESM2_ESM.tif]
